# A Self-Calibrating IoT Portable Electrochemical Immunosensor for Serum Human Epididymis Protein 4 as a Tumor Biomarker for Ovarian Cancer

**DOI:** 10.3390/s20072016

**Published:** 2020-04-03

**Authors:** Valentina Bianchi, Monica Mattarozzi, Marco Giannetto, Andrea Boni, Ilaria De Munari, Maria Careri

**Affiliations:** 1 Department of Engineering and Architecture, University of Parma, 43124 Parma, Italy; valentina.bianchi@unipr.it (V.B.); andrea.boni@unipr.it (A.B.); ilaria.demunari@unipr.it (I.D.M.); 2 Department of Chemistry, Life Sciences and Environmental Sustainability, University of Parma, 43124 Parma, Italy; maria.careri@unipr.it

**Keywords:** human epididymis protein 4, competitive electrochemical immunosensor, WiFi portable potentiostat, on-board calibration, Internet of Things

## Abstract

Nowadays, analytical techniques are moving towards the development of smart biosensing strategies for the point-of-care accurate screening of disease biomarkers, such as human epididymis protein 4 (HE4), a recently discovered serum marker for early ovarian cancer diagnosis. In this context, the present work represents the first implementation of a competitive enzyme-labelled magneto-immunoassay exploiting a homemade IoT Wi-Fi cloud-based portable potentiostat for differential pulse voltammetry readout. The electrochemical device was specifically designed to be capable of autonomous calibration and data processing, switching between calibration, and measurement modes: in particular, firstly, a baseline estimation algorithm is applied for correct peak computation, then calibration function is built by interpolating data with a four-parameter logistic function. The calibration function parameters are stored on the cloud for inverse prediction to determine the concentration of unknown samples. Interpolation function calibration and concentration evaluation are performed directly on-board, thus reducing the power consumption. The analytical device was validated in human serum, demonstrating good sensing performance for analysis of HE4 with detection and quantitation limits in human serum of 3.5 and 29.2 pM, respectively, reaching the sensitivity that is required for diagnostic purposes, with high potential for applications as portable and smart diagnostic tool for point-of-care testing.

## 1. Introduction

Ovarian carcinoma (OC) is the leading cause of death from gynecological malignancy worldwide: it is generally asymptomatic in the early stage, so the majority of women with OC are not diagnosed until the disease is in an advanced stage, with an overall five-year relative survival rate that is generally in the 30–40% range [1,2]. Therefore, it is crucial to detect OC as early as possible to correctly identify the cancer stage for effective treatment options and to early distinguish malignant from benign pelvic mass by means of annual routine gynecological and pelvic examinations, as well as dedicated screening programmes. In this context, noninvasive cancer detection at an early stage needs the identification of specific and sensitive biomarkers that are present at abnormal concentrations in body fluids, as well as the development of smart devices for analytical screening [3,4,5,6], in order to improve ovarian cancer survival rate [7]. Currently, carbohydrate antigen 125 (CA125) glycoprotein is the established biomarker for detecting OC occurrence and the monitoring therapeutic progress. However, the diagnostic potential of CA125 is mainly limited by its low specificity, since it can also be increased in a variety of other benign gynecological conditions affecting pre- and/or post-menopausal women, or in other malignant cancers [8]. In this context, over the last years, a number of alternative OC biomarkers, especially for the predominant OC subtype, i.e. epithelial ovarian cancer (EOC), have been identified and studied, either alone or combined, to improve the effectiveness of early diagnostic strategies. Among the recently proposed biomarkers, human epididymis protein 4 (HE4) resulted in being most promising in EOC differential diagnosis in detecting the disease at early stage, in monitoring the response to chemotherapy and in estimating the prognosis of ovarian cancer [1].

Electrochemical immunosensors are playing a growing role for improved clinical diagnosis and therapy monitoring by combining the specificity of antigen-antibody immunoreaction and the sensitivity of the electrochemical transduction [9]. As for HE4 determination, several sensing architectures that are based on “sandwich” approach have been recently exploited [10,11,12,13].

Electrochemical immunosensors can exploit enzyme labels for signal generation, such as the here presented study, but can also be designed according to label-free transduction and amplification mechanisms [14,15]. One of the main advantages associated to the enzyme-labelled electrochemical transduction is the possibility of method implementation on miniaturized electrodes as well as on portable and battery-operated devices [16]. Several instrumental approaches have been proposed in recent years and, some of these, such as 910 PSTAT and DropStat from Metrohm (Herisau, Switzerland), are already commercially available. DropStat, in particular, allows for making quantitative analyses and directly displaying the analyte concentration on the stand-alone device; the tool is custom configured with the electrochemical technique attending user’s needs by simply inserting preprogrammed cards with calibration parameters available upon request from the producer. A PC connection is necessary to download acquired data for storage purposes. In addition, solutions by PalmSens (Houten, The Netherlands) and UNISCAN (Buxton, Derbyshire, UK) are battery-powered or USB-powered portable devices that are capable of performing voltammetric measurements, but they need to rely on external APP or software PC to determine the analyte concentration in a sample. Very recently, USB-based [17,18,19] and wireless [20,21,22] potentiostats were proposed, both involving data processing and visualization performed on an external device running a custom developed software. A Bluetooth communication for data collecting, processing, and sharing is exploited, even in the case of wireless potentiostats, thus requiring a PC that is close to the device. In 2015, Steinberg et al. proposed a device based on RFID or NFC data communication, where data are logged and then off-line analyzed in MS-Excel format [23]. However, all of the developed systems that allow for quantitative analyses to be performed involve off-line data processing based on a calibration function, even if using a Wi-Fi communication protocol. For example, Annamalai and coworkers recently described a Wi-Fi based solution for glucose determination, taking advantage from the Adafruit Cloud services to process and display data [24]. Chen et al. reported another recent example that involves Wi-Fi protocol that can locally visualize results on a TFT LCD display [25].

In this context, the possibility to perform data processing and calibration on-board, making the device completely autonomous, is very attractive. This approach allows, on the one hand, to reduce the device power consumption avoiding the raw data transmission and, on the other hand, to increase its portability, making it usable independently of connections or external devices at the time of measurement. However, the potentiostat portability must not affect the measurement accuracy in estimating the analyte concentration. Very recently, our research group proposed a new portable potentiostat having the Analog Front End (AFE) that was capable of very accurate analysis when compared to other portable solutions that are available in literature [26]. In this context, the present work represents a further improvement of that Wi-Fi portable potentiostat, proposing new additional functions through an enhanced firmware to introduce autonomous calibration and processing capabilities with the aim of applying it to the readout and processing of data from an innovative magneto-immunoassay for the determination of the ovarian cancer biomarker HE4 in human serum. This approach is consistent with the technological evolution of Analytics 4.0, representing the innovative integration of analytical chemistry into an Internet-of-Things (IoT) system, as recently defined by Mayer at al. [27].

## 2. Materials and Methods

### 2.1. Materials

Trizma^®^ base, Tween-20, boric acid, sodium hydroxide, monosodium phosphate, disodium phosphate, magnesium chloride, human serum, albumin from bovine serum (BSA), Monoclonal Anti-WFDC2 clone 3F9 Antibody produced in mouse purified immunoglobulin (anti-HE4), PrEST Antigen WFDC2 (HE4) were purchased from Sigma Aldrich (Milan, Italy). Alkaline Phosphatase-conjugated secondary Rabbit Anti-Mouse IgG (RAM-AP), carcinoembryonic antigen (CEA), and Carbohydrate Antigen 125 (CA125) were purchased from Abcam (Cambridge, UK). DropSens^®^ hydroquinone diphosphate (HQDP) was obtained from Metrohm Italiana (Origgio, Varese, Italy). The following buffers were prepared: 0.1 M borate buffer (BB) pH 9.5; 0.1 M Na-phosphate buffer (PB; 0.08 M Na_2_HPO_4_ and 0.02 M NaH_2_PO_4_) pH 7.4; 0.1 M TRIS Buffer (TB) containing 0.02 M of MgCl_2_ pH 7.4; washing buffers PB-T and TB-T consisted, respectively, of PB and TB containing Tween-20 at 0.05 % (*v/v*). Reading buffer (RB) was prepared as TB, but adjusting the pH to 9.8.

Dynabeads^®^ M-280 Tosylactivated (2.8 µm) MBs were purchased from Invitrogen (Thermo Fisher Scientific, Rodano, Milan, Italy). DropSens^®^ Screen-Printed Carbon Electrodes (SPEs) for electrochemical readout were purchased from Metrohm Italiana; in particular, the working (WE; 4 mm diameter) and auxiliary (AE) electrodes are made of carbon, while pseudo-reference electrode (RE) is in silver.

### 2.2. Magnetic Immunoassay Set-Up

MBs were thoroughly suspended and a volume of 15 μL was transferred to a new tube, then, after triple-washing step with 200 μL of BB, the MBs were incubated with 400 μL of 10 μg mL^−1^ solution of HE4 antigen in BB (1 h; 37 °C; 1000 rpm). After the removal of unreacted HE4 solution by twice washing with BB, MBs were blocked with a 2% (*w/v*) solution of BSA dissolved in PB, and then washed with PB-T and PB. The HE4-modified MBs were used to carry out four independent competitive electrochemical immunoassays after their resuspension in 400 μL of TB and subdivision in four equivalent volume aliquots. After isolation, the MBs were resuspended with 100 μL of serum sample previously diluted ten-fold with TB and added with anti-HE4 monoclonal antibody to reach the final concentration of 1 μg mL^−1^. The immunocompetition was left to undergo for 1 h at room temperature (RT) under agitation (1000 rpm), the reacted MBs was carefully washed with TB-T and TB and then incubated with 100 μL of 2 μgmL^−1^ RAM-AP secondary antibody (RT; 1 h; 1000 rpm). After washing with TB-T and TB, the beads were finally reacted with 100 μL of 1 mg mL^−1^ solution of HQDP enzyme substrate dissolved in RB for three minutes. Two 50 μL sub-aliquots of each sample were transferred on a SPE mounted on a DropSens^®^ customized magnetic support that aimed at confining the MBs on the surface of WE and the electrochemical readout was carried out independently for each sub-aliquot of the sample suspension. Alkaline phosphatase (AP) dephosphorylates non-electroactive HQDP into hydroquinone (HQ), allowing for its electrochemical oxidation to quinone (Q) to achieve the signal related to the analyte. 

**Scheme 1:**
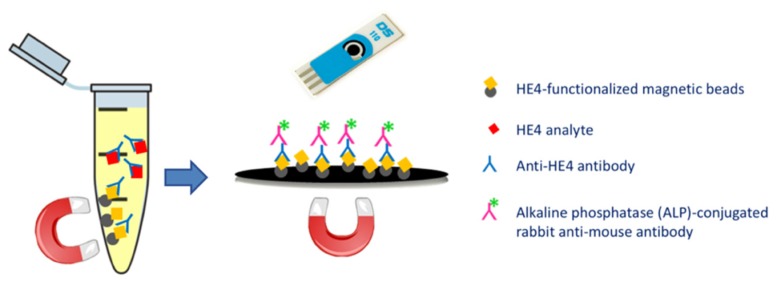
Working principle of the electrochemical immunomagnetic assay on MBs supports.

Scheme 1 reports a schematic representation of the working principle of the electrochemical immunomagnetic assay.

The voltammetric readout of the assays were performed through our homemade IoT Wi-Fi portable potentiostat, while using the differential pulse voltammetry (DPV) scan. The potential was scanned between −0.4 and +0.2 V, setting on the IoT potentiostat a pulse amplitude of 0.05 V, a step potential of 0.005 V, and a pulse time of 100 ms. After the deposition of the MBs suspension on the SPE, a preconditioning step of 30 s at −0.4 V was applied to preconcentrate the reduced form of quinone. 

The current responses observed for each concentration (S) were normalized with respect to the zero signal (S_0_) obtained without HE4 in competition. 

### 2.3. IoT Architecture

**Scheme 2:**
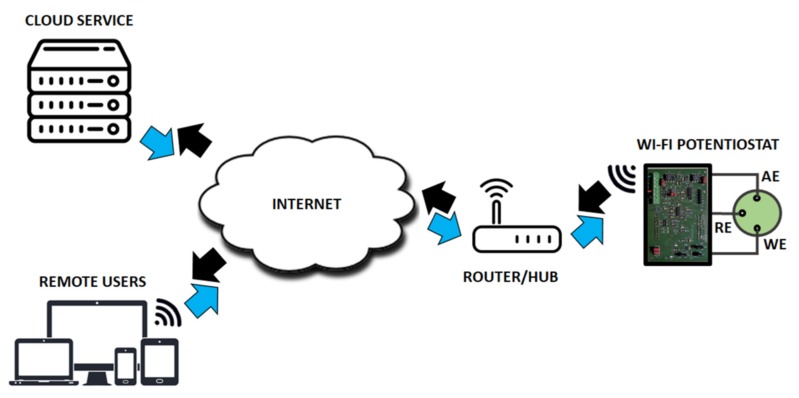
Internet-of-Things (IoT) architecture for the proposed system.

The system architecture is depicted in the sketch that is shown in Scheme 2. 

The exploited AFE guarantees a sufficient measurement points to achieve higher accuracy with respect to other solutions presented in literature, as reported in our previous study [26]. In this work, the aforementioned AFE is connected to a CC3200 Microcontroller Unit (MCU) for signal processing with an improved firmware to develop a smart self-calibrating portable potentiostat, that was fully compliant with the IoT paradigm. 

In particular, the proposed system takes a step forward with respect to current literature [17,18,19,20,21,22,23,24,25], since it is capable of on-board building a calibration function acquiring the signal from a given set of standards at known concentrations (*calibration mode*); this function is subsequently applied to calculate analyte concentration of unknown samples (*measurement mode*). Moreover, the proposed solution relies on a Wi-Fi connection to send the preprocessed data to a cloud service for storing and sharing exploiting the remote access feature. The on-board data elaboration allows for greatly reducing the device power consumption, enhancing battery lifetime. In fact, the main contribution to the device power consumption is the data transmission phase: sending preprocessed information allows to reduce the transmission task, with a benefit for the average absorbed current. In Bassoli et al. [28], a quantitative evaluation of this approach in the case of Wi-Fi protocol is reported. The data generated from the end device go directly, through the modem/router via Wi-Fi Protected Access (WPA) standard protocol, to the Internet cloud, where they are stored for subsequent sharing in a fast and efficient way. Thanks to the broad coverage guarantee by the Wi-Fi router, it is easy to ensure a stable sensor connection over a large area [29]. The scenario is fully compliant with an IoT paradigm, in which sensors “information flows rationally and orderly on the Internet, for being shared on a global scale” [30]. 

### 2.4. On-Board Data Management

The basedrift phenomenon has to be considered to correctly estimate the DPV peak current. In fact, the differential current baseline is often not parallel to the voltage axis, which prevents the computation of the peak by simply applying an algorithm for the search of the maximum. Hence, for each measurement point a baseline estimation algorithm is applied. Usually, for each curve, N minimum points have to be detected and the baseline is evaluated applying a polynomial fitting algorithm. Therefore, the baseline thus calculated is assumed as the reference for the correct evaluation of the peak current. 

For calibration purposes, the peak current values have to be related to the HE4 concentration according to a suitable mathematical law. For this purpose, the normalized *S*/*S*_0_ responses were plotted versus the HE4 concentration, so obtaining a dose-response inhibition curve, which was interpolated through a four-parameter logistic function (1) [31]:

(1) $$S/S_{0}=S_{min}+\frac{\left(S_{max}-S_{min}\right)}{1+{\left(\left[C\right]/I_{50}\right)}^{B}}\;,$$

where *S_min_* and *S_max_* are the asymptotic minimum and maximum, *I_50_* is the inflection point, namely the antigen concentration corresponding to 50% signal inhibition and *B* is the slope at *I_50_*.

In the *calibration mode*, the device is able to compute on-board these four parameters when considering a set of measurements that were carried out on calibration standards. In particular, the parameters are computed, implementing the method of least squares according to the Levenberg–Marquardt algorithm [32,33]. When considering two vectors *X* and *Y* in *Rn*, containing, respectively, the *n* known concentration values and the *n* measurements of the corresponding peak current to be used for calibration, the vector of the residuals *ε* in *Rn* will be defined, as

(2) ε=Y−F(X, β),

where *F* is the logistic function and *β* is the vector containing the four parameters to be computed. Hence, the aim of the proposed algorithm is to minimize the sum of the residual squares, *SS*, with an iterative approach. In doing so, the first step is to define the initial values for the parameters to be found, which are

(3) $$S_{\max\_0}=\max\left(Y\right),$$

(4) $$S_{\min\_0}=\min\left(Y\right),$$

(5) $$B_{0}=sign\;\left(\frac{y_{n}-y_{1}}{x_{n}-x_{1}}\right)\;,$$

(6) $$I_{50\_0}=X_{k},\;\;\;\;\;\;k\;\mathrm{is}\;\mathrm{the}\;\mathrm{position}\;\mathrm{where}\;\min\;\left(\left|Y-\frac{\max\left(Y\right)-\min\left(Y\right)}{2}\right|\right),$$

Subsequently, during a generic step *i* the *β* vector is updated as

(7) $$\beta_{i}=\beta_{i-1}-\Delta_{\beta_{i-1}}\;\;,$$

where Δ*_βi_* is defined as

(8) $$\Delta_{\beta_{i}}={\left({\left(J^{T}J\right)}_{\beta_{i}}+\lambda {\left(J^{T}J\right)}_{\beta_{i}}\right)}^{-1}J^{T}e_{\beta_{i}}\;\;,$$

*λ* is a parameter updated at each step and *J* is the Jacobian matrix defined as

(9) $$J=\;\frac{\partial e}{\partial \beta }\;\;,$$

Finally, the sum of the residual square is computed as

(10) $$SS_{i}=\;\sum_{i}{\left(\varepsilon_{i}\right)}^{2}=\varepsilon_{\beta_{i}}^{T}\varepsilon_{\beta_{i}}\;\;,$$

If *SS_i_* is equal to *SS_i−1_* with a chosen tolerance of 1 × 10^−6^, convergence is reached and the algorithm stops. Otherwise, the *λ* parameter is updated according the following law (11) and the algorithm continues.

(11) $$\lambda_{i+1}=\{\begin{array}{c} 0.1\lambda_{i}\;if\;SS_{i}<SS_{i-1}\\ 10\lambda_{i}\;otherwise \end{array}\;\;\;,$$

All of these operations were coded in C language for MCU programming and specific function to compute the derivative of residual with respect to each parameter in *β* vector was developed in order to compose the Jacobian matrix J.

Switching to the *measurement mode*, the inversion of the calibration function involves the computation of a *B*-th root. This type of calculation is trivial if it is performed on a platform with a high computing capacity, like the one made available by the main cloud services. However, processing the data in the cloud implies sending all of the collected raw data through the Wi-Fi energy-intensive connection, considerably increasing the power consumption and limiting the portability of the device. Processing data on board limits these issues, but, on the other hand, it entails fewer computational resources and the need to develop specific functions in C language. To implement the *B*-th root on the MCU platform, a generalization of the Heron’s method was exploited [34]. Once the concentration has been computed, it can be made available on a cloud for remote monitoring or can be maintained in the device memory waiting a Wi-Fi connection.

### 2.5. Immunosensor Validation

The validation of sensing device was performed according to the Eurachem guidelines [35] on human serum as blank matrix. An assessment of the dose-response calibration curve was carried out using concentration values already corrected according to the serum dilution factor. 

The detection (LOD) and quantitation (LOQ) limits were calculated from mean normalized signal ($y_{b}$*)* and standard deviation ($s_{b}$) recorded from ten independent replicates at HE4 level showing a not significant signal inhibition (35 fM, first point of dose-response curve, see 3.4 paragraph). More precisely, the corresponding signals were calculated, as $y_{b}-\left(3.3\cdot \left(s_{b}/\sqrt{n}\right)\right)$ and $y_{b}-\left(10\cdot \left(s_{b}/\sqrt{n}\right)\right)$, respectively, where *n* is the number of independent replicates for each concentration level. The linear dynamic range was determined as the concentration interval over which the slope (*B* parameter of the inhibition curve) varies within ±10%. Two concentration levels that were not explored for the calibration were used for the assessment of trueness.

## 3. Results and Discussion

### 3.1. MBs Functionalization and Immunocompetition Study

The competitive magneto-immunoassay was implemented on tosyl-activated magnetic microbeads as sensing substrate, involving the lysine residues of HE4 antigen for its covalent linking. The immobilization of the antigen is one of the most critical issues when developing immunoassays, as this procedure has to not compromise the immunoreactivity of the bioreceptor, i.e., not involving the antibody-binding regions.

Before carrying out the competition experiments, the maintenance of HE4 immunoreactivity was verified through direct binding titrations between the immobilized antigen and anti-HE4 antibody. These experiments were also aimed at investigating the dynamic range for the concentrations of (*i)* HE4 used for MBs functionalization and (*ii*) anti-HE4 used for the implementation of the immunocompetition. For this purpose, we fixed at 10 μg mL^−1^ the HE4 concentration for the MBs functionalization and, thus, we incubated the modified beads with anti-HE4 ranging from 0 to 2 μg mL^−1^, and then with RAM-AP secondary antibody. Further experiments were carried out varying the antigen concentration from 0 to 20 μg mL^−1^, keeping the anti-HE4 concentration at 2 μg mL^−1^ constant. The results of the immuno-titration experiments showed current responses increasing versus anti-HE4 and HE4 concentrations, with very low background signals recorded in the absence of antibody and antigen.

These findings can be rationalized as an evidence of the effective antigen immobilization, also being confirmed by the non-occurrence of unwanted non-specific binding on MBs surface. 

**Figure 1:**
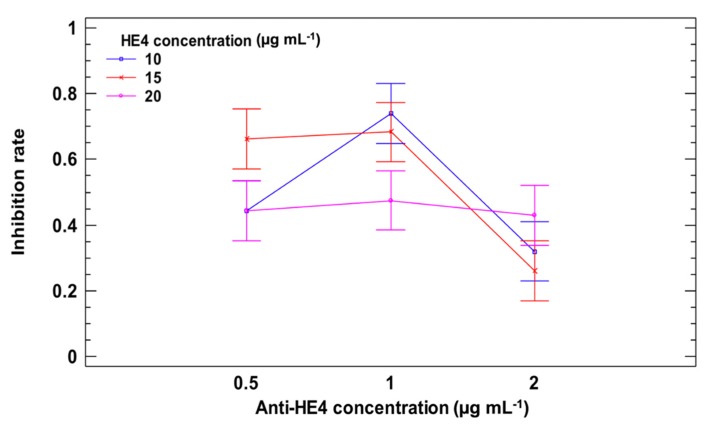
Interaction plot from two-way ANOVA. Factors: concentration of HE4 for functionalization and concentration of anti-HE4 antibody; Response: signal inhibition.

On the basis of the above considerations, we performed a set of competition experiments keeping constant at 10 μg mL^−1^ the competing HE4 and varying the above discussed experimental parameters. The dataset of signal inhibition values, being calculated as 1-(*S*/*S_0_*), was evaluated through a two-way Analysis of Variance (ANOVA), showing that the anti-HE4 factor and its interaction with HE4 factor significantly influence (*p* < 0.01) the immunosensor response, whereas the single HE4 factor resulted in being not significant (*p* > 0.01). Figure 1 reports the ANOVA interaction plot, showing the effect of the two studied factors. The observed trend clearly shows the higher signal inhibition rate being observed for the combination of the middle (1 μg mL^−1^) anti-HE4 concentration level and the lowest (10 μg mL^−1^) HE4 concentration explored for the functionalization of MBs. The best concentrations for the immunocompetition experiments were then chosen on the basis of these results. 

As for functionalized MBs stability, intended as the maintenance of the reactivity of HE4 immobilized on the magnetic beads, it resulted in being good over at least two months stored in the dark at +4 °C.

### 3.2. Data Acquisition and Processing

**Figure 2:**
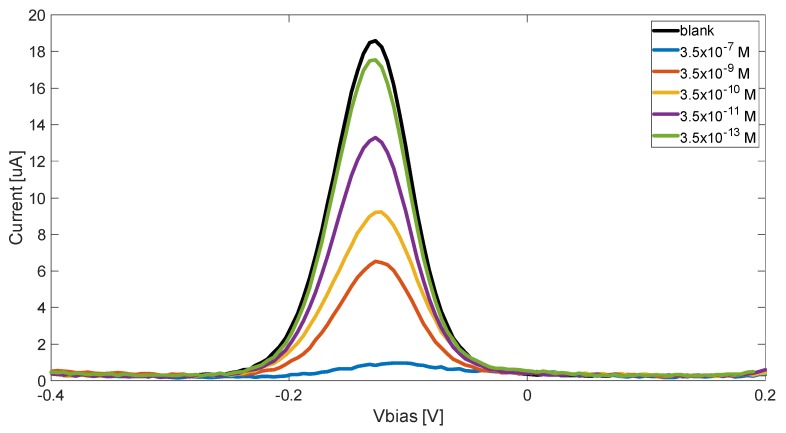
Differential pulse voltammetry (DPV) scans recorded carrying out immunocompetition assays over the 350 fM-350 nM HE4 concentration range in human serum samples.

A *Vbias* ranging from −0.4 V to 0.2 V, which was suitable for acquisition of HQ oxidation current, was applied between WE and RE electrodes of the cell to measure the amperometric signal (i.e., the cell output current). Exploiting the high resolution of the AFE reported in our previous work [26], a total of 120 measurement points of differential output current were collected. Figure 2 shows the signals that were recorded over the 350 fM–350 nM HE4 concentration range. 

**Figure 3:**
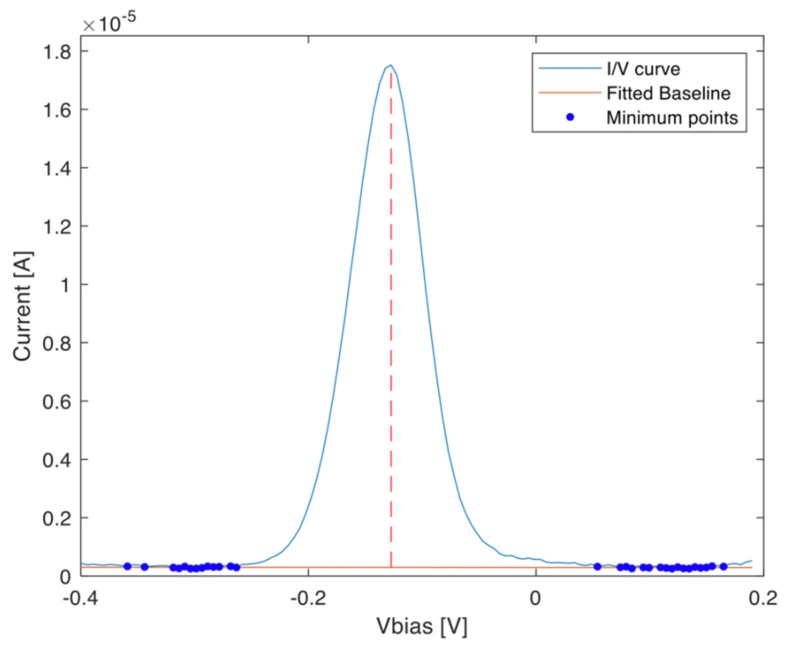
Example of baseline estimation from a voltammogram. Thirty minimum points were considered for the linear regression.

In this application, a minimum number of point equal to 30 was set for baseline drift correction. Figure 3 illustrates an example of baseline estimation in the case of 350 fM HE4 concentration. The dots indicate the 30 minimum points that were exploited in the linear regression and the resulting baseline is shown. The actual peak value is calculated as the distance between the baseline and maximum current value.

**Figure 4:**
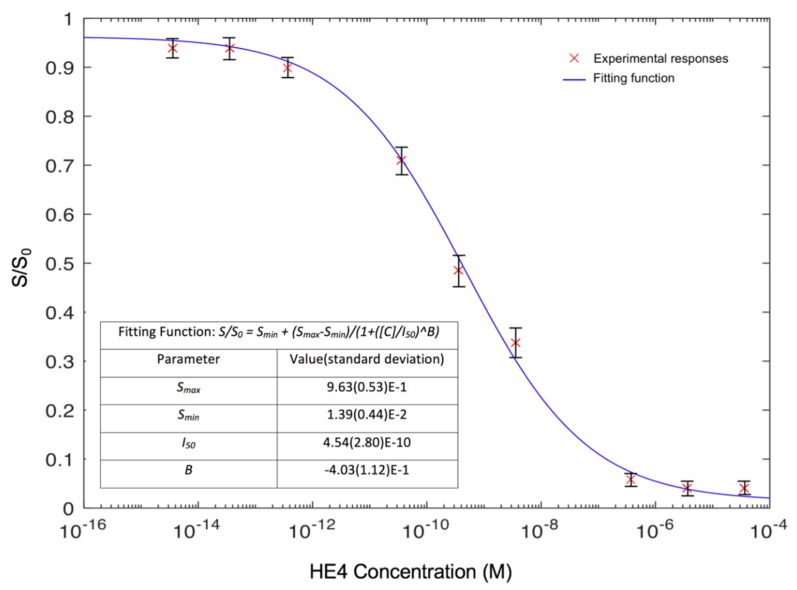
Dose-response inhibition curve for HE4-spiked human serum samples obtained interpolating the dataset from immunocompetition experiments carried out on HE4-modified MBs over the 3.5 fM–35 μM concentration range while using the four-parameter logistic function (1). Inset table: fitted curve parameters.

Figure 4 shows the dose-response inhibition curve that was obtained upon interpolation of the experimental dataset with the four-parameter logistic function (1). An increase of analyte concentration was found to reduce the amount of anti-HE4 that was bound to the antigen-modified MBs, thanks to the ability of HE4 to inhibit the antibody binding extent to the sensing surface that was functionalized with the antigen.

### 3.3. Electrochemical Immunoassay Analytical Performance

The developed IoT immunochemical device exhibited good analytical performance showing a linear dynamic range over three orders of magnitude. As for precision assessed on the basis of three independent assays, we observed relative standard deviations (RSD) always < 13%. 

The analysis of HE4-spiked serum samples provided a LOD of 3.5 pM and a LOQ of 29.2 pM. A comparison of the calculated LOD with the threshold values set for premenopausal women (70 pM) and postmenopausal patients (140 pM) [36] highlights the suitability of the developed system in terms of quality criteria for routine analysis, while also considering the high automatization level in calibration and data processing and the assessment of all validation parameters in matrix, differently from previously published studies.

Trueness was assessed while using HE4-spiked serum samples with concentration levels that were not used for the calibration, i.e. LOQ and *I_50_*, obtaining a mean recovery of 105 ± 12%. These values can be considered to be very satisfying while taking the developed on-board self-calibration innovative strategy, embedded in the portable automatized instrument, into account. 

### 3.4. Selectivity Assessment

Protein markers potentially occurring in clinical samples of diagnostic interest for carcinomas of gynecologic concern were selected to assess the selectivity of the developed immunomagnetic assays. For this purpose, we evaluated the responses that were obtained with CA125 and CEA, both at *I_50_* concentration levels, obtaining no significant signal inhibition (*p* < 0.05) with respect to blank reference samples only containing anti-HE4 antibody. 

## 4. Conclusions

In the present work, we proposed an innovative approach to address disease diagnosis and management through the integration of IoT home-made portable potentiostat with electrochemical immunosensors for the determination of HE4 as ovarian cancer serological biomarker in the framework of the point-of-care (POC) testing. The choice of a mainstream protocol for the connection as Wi-Fi enhances the usability and portability of the system without requiring an additional device, such as PC or Smartphone. Thanks to the ability of the developed portable device to acquire a high number of measurement points (i.e. 350), an accurate quantitative evaluation of the analyte concentration can be carried out. The developed device is able to perform the interpolation of calibration function and the concentration evaluation directly on-board, enhancing the system portability, minimizing data transmission, and therefore the high power consumption that is generally required by the Wi-Fi connection. In the case of absence of connectivity, the device can store the data in a local memory waiting for an available wireless connection.

The analytical performance of the IoT-based competitive immunomagnetic electrochemical assay was assessed in human serum, obtaining LOD and LOQ values at the picomolar level, meeting the requirements for diagnostic applications in early diagnosis of OC. Finally, the sensing device was successfully applied in serum with adequate recovery values for the analysis of spiked samples as well as good selectivity toward potential protein interferents. This work shows great promise for the application of the developed self-calibrating IoT portable immunosensor for HE4 determination in serum samples, in accordance with Analytical 4.0 evolution towards smart platforms for decentralized POC testing.
